# Resolving Fine-Scale Heterogeneity of Co-seismic Slip and the Relation to Fault Structure

**DOI:** 10.1038/srep27201

**Published:** 2016-06-03

**Authors:** C. W. D. Milliner, C. Sammis, A. A. Allam, J. F. Dolan, J. Hollingsworth, S. Leprince, F. Ayoub

**Affiliations:** 1 Department of Earth Sciences, University of Southern California, Los Angeles, California 90089, USA; 2 Geology and Geophysics, The University of Utah, Salt Lake City, Utah 84112, USA; 3 Université Grenoble Alpes, ISTerre, Grenoble 38058, France; 4 Navdy, 575 7th Street San Francisco, CA 94103, USA; 5 Division of Geological and Planetary Sciences, California Institute of Technology, Pasadena, California 91125, USA

## Abstract

Fault slip distributions provide important insight into the earthquake process. We analyze high-resolution along-strike co-seismic slip profiles of the 1992 M_w_ = 7.3 Landers and 1999 M_w_ = 7.1 Hector Mine earthquakes, finding a spatial correlation between fluctuations of the slip distribution and geometrical fault structure. Using a spectral analysis, we demonstrate that the observed variation of co-seismic slip is neither random nor artificial, but self-affine fractal and rougher for Landers. We show that the wavelength and amplitude of slip variability correlates to the spatial distribution of fault geometrical complexity, explaining why Hector Mine has a smoother slip distribution as it occurred on a geometrically simpler fault system. We propose as a physical explanation that fault complexity induces a heterogeneous stress state that in turn controls co-seismic slip. Our observations detail the fundamental relationship between fault structure and earthquake rupture behavior, allowing for modeling of realistic slip profiles for use in seismic hazard assessment and paleoseismology studies.

The spatial pattern of fault slip associated with large earthquakes provides fundamental insight into rupture mechanics^1^, faulting processes^2^ and seismic radiation^3^. However, our current understanding of co-seismic surface slip distributions is limited by the inherent drawbacks of standard measurement methodologies: geodetic data are typically either too sparse to resolve fine-scale heterogeneity (e.g., point Global Positioning System measurements), or adversely affected by strong shaking and deformation near the surface rupture (e.g., loss of phase coherence in Interferometric Synthetic Aperture Radar data)^4^, whereas geologic field measurements typically cannot include off-fault deformation^5^ and are confined to sparse along-fault locations^6^,^7^. Co-seismic slip as constrained by these coarse data has been well-characterized to first order as smooth, semi-elliptical or triangular distributions with fluctuations related to seismogenic-scale fault segmentation, or off-fault yielding^1^,^8^,^9^. However, these first-order models fail to explain observations from more recent higher-resolution field and geodetic studies, which have consistently documented high-amplitude, short-wavelength variation of slip, even when incorporating the effects of off-fault deformation^10^,^11^,^12^,^13^,^14^. Thus, several key questions remain: Why does the short-wavelength variability exist? Does this variability follow any pattern? And what is the physical explanation for this variability?

In this study, we address these questions by generating high-resolution along-strike co-seismic slip profiles for the 1992 M_w_ = 7.3 Landers and 1999 M_w_ = 7.1 Hector Mine earthquakes using subpixel optical image correlation^15^. These data allow for the first time an analysis of the frequency content of co-seismic slip along an entire surface rupture with high density and number of measurements to high precision. We find from analyzing the fractal properties of the slip distribution and fault structure, that the along-strike slip variability correlates with zones of geometrical fault complexity at all scales. From this correlation we infer that fault complexity gives rise to heterogeneity in the stress field, which in turn causes variable co-seismic slip.

The 1992 M_w_ = 7.3 Landers and 1999 M_w_ = 7.1 Hector Mine earthquakes are ideal candidates for investigating and directly comparing co-seismic deformation patterns between two events for several reasons. First, the two events occurred on kinematically similar NNW-trending right-lateral fault systems only 20 km apart from one another, and are located within the same tectonic regime, the Eastern California Shear Zone, (see inset map in Fig. 1a). Second, pre- and post-event high-resolution aerial photographs of 1 m pixel size exist for both earthquakes acquired from the same optical sensor with similar flight parameters (see Methods). Finally, both ruptures extend through an arid desert region providing optimal conditions for subpixel correlation between image pairs acquired at different times as surface features are well preserved, and also the surface ruptures are not obscured by either vegetation or urban development. Thus, these conditions allow for complete constraint of the near-field deformation in high-resolution, with the use of data of comparable spatial resolution and accuracy between the two earthquakes.

## Results

### Optical image correlation

**Figure 1:**
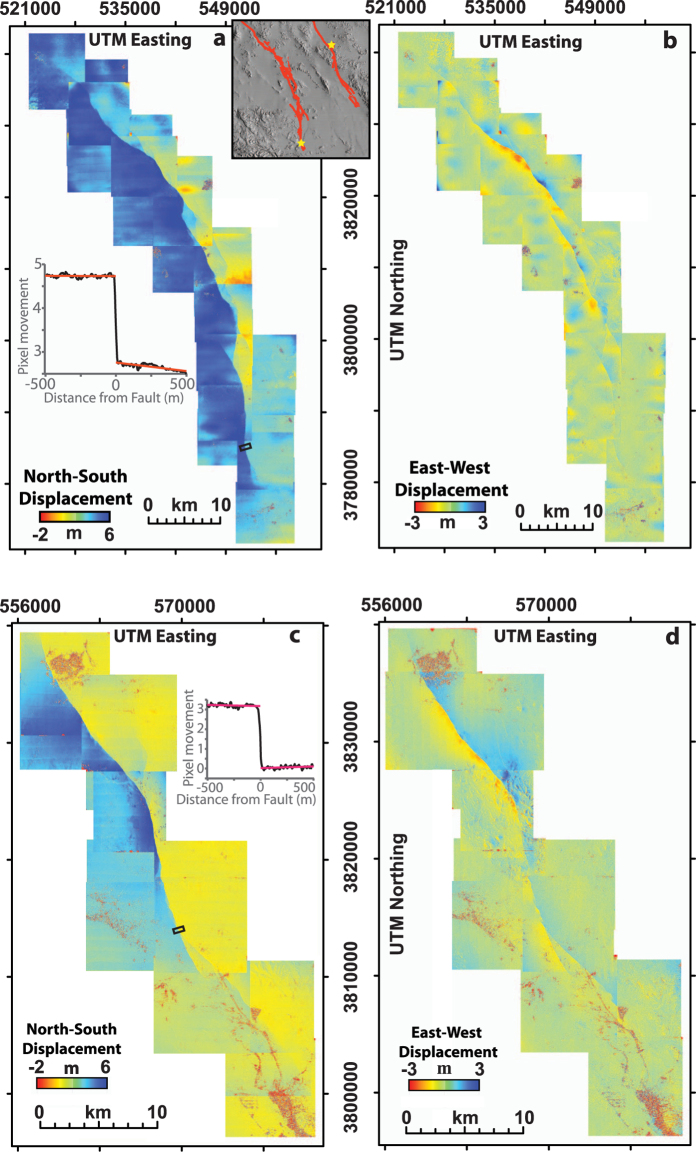
Correlation maps for the 1992 Landers and 1999 Hector Mine earthquakes. Deformation maps showing north-south (**a,c,** left column) and east-west (**b,d,** right column) component of displacement with positive values indicating movement to the north and east, respectively for the (**a,b,** top) Landers, and (**c,d,** bottom) Hector Mine events. The inset map in **a**, top right, shows the regional location of the 1992 Landers^28^ and 1999 Hector Mine^29^ rupture in red lines overlaid onto a hillshade10 m national elevation dataset digital elevation model, with yellow stars denoting the location of the epicenter. The inset figures in (**a,c**) show fault-parallel displacement (black line) within a 138 m wide stacked profile (also shown on the correlation maps as a black rectangle), illustrating how fault offset is measured using linear regressions (red lines), which are manually fit to the deformation signal on either side of the fault. A total of 1081 profiles were measured for Landers and 470 for Hector Mine; these measurements were compiled along-strike to create the co-seismic slip profiles shown in Fig. 2a,b. The displacement maps were computed using COSI-Corr and plotted within ENVI 4.8 (http://www.exelisvis.com/ProductsServices/ENVIProducts/ENVI.aspx) and Arcmap 10.1 (http://www.esri.com/software/arcgis/arcgis-for-desktop). Air photo data compiled by the U.S. Geological Survey (http://www.usgs.gov).

**Figure 2:**
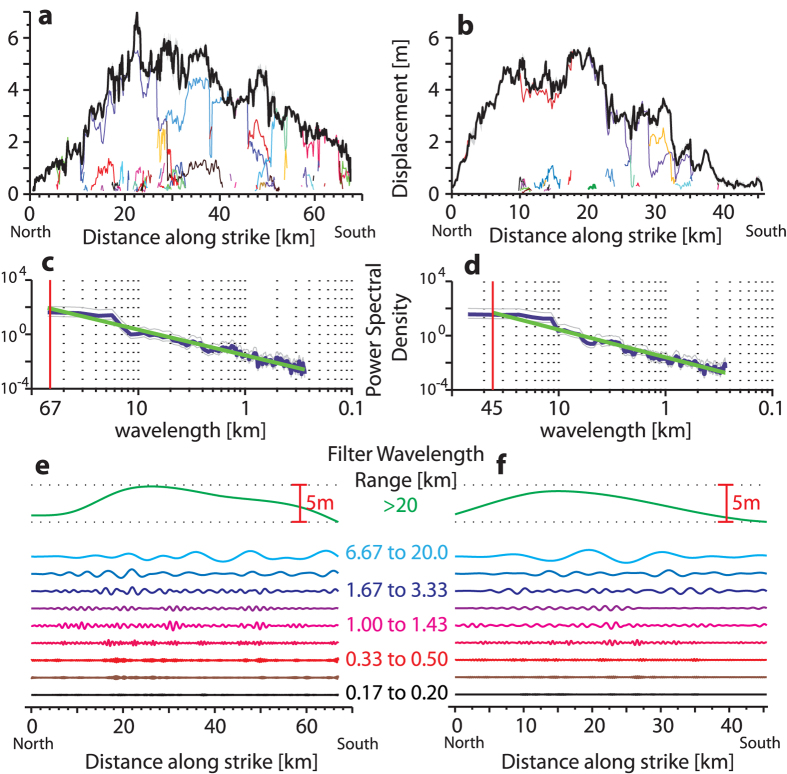
Analysis of co-seismic slip variation. (top) Slip profiles for (**a**), Landers, and (**b)**, Hector Mine, shown by the black line. (middle) Power spectral density of the slip profiles for (**c)**, Landers, and (**d),** Hector Mine. Both profiles follow fractal distributions with no characteristic frequencies, red line delineates the corner frequency which is determined by the rupture length (which the fundamental mode cannot exceed), light gray lines denote the 95% confidence interval on the estimate of the power spectrum and green line the linear least-squares regression with coefficient of determination (R^2^) of 0.92 and 0.94 for (**c,d)** respectively. (**e,f)** Band-passed components of slip profiles at specific wavelengths, vertical red scaling bar defines the amplitude of slip, labels on y-axis denote the range of wavelength cut-off values used to filter the slip distribution at specific bands, with arbitrary vertical spacing for illustrative purposes. The band-pass filtering of slip at specific frequencies illustrates how its amplitude progressively diminishes with shorter wavelengths, a characteristic of a self-affine fractal distribution. Filters were performed with a Butterworth filter^23^, see Methods for more information on the specific upper and lower corner wavelength values used for each band.

We measured co-seismic fault slip for the two events using the optical image correlation software COSI-Corr (Co-registration of Optically Sensed Images and Correlation)^15^, which allows for the precise co-registration, orthorectification and subsequent correlation of pairs of pre- and post-event aerial photographs (see Methods for detailed processing steps). Specifically, to quantify surface motion we track movement of features between pairs of pre and post-event ortho-air photos using COSI-Corr’s phase correlator that uses an iterative, unbiased processor that estimates the phase plane in the Fourier domain^15^. The correlation results produced from subpixel matching of the before and after air photos are presented in terms of 2D horizontal surface deformation maps (Fig. 1). These reveal the spatial distribution of near-field co-seismic surface deformation as small as 10 cm along multiple fault strands that are consistent between overlapping image pairs and with almost no decorrelation. Geometrical artifacts in the correlation results yield metric biases, which can be observed as horizontal ‘streaks’ in Fig. 1 caused by scanning distortion and thermo-mechanical warping of the film, but are limited to wavelengths (>1 km) significantly larger than the deformation signal associated with the width of the earthquake rupture (<100 m)^16^,^17^. Furthermore, a series of synthetic tests have confirmed that these long-wavelength artifacts exert no influence on our measurements of fault displacement (See Methods and Supplementary Fig. S2–7), in agreement with previous studies^16^,^17^. From the correlation maps, we measured the right-lateral displacement at points along the surface rupture using 1–3 km-long, 138 m-wide stacked, fault-normal profiles (dimensions determined from synthetic tests see Methods and Supplementary Information). We note, the complete constraint of the near-field deformation pattern allows for measurement of both localized on-fault deformation and distributed off-fault deformation, meaning each ‘displacement’ measurement represents the total fault-parallel deformation accommodated across the entire fault zone. By making 1551 such measurements (1081 for Landers and 470 for Hector Mine) at a uniform along-strike spacing of 138 m, we constructed co-seismic slip distributions (Fig. 2a,b) using the largest number of samples ever reported in a single study. We note the maximum amplitude of post-seismic afterslip observed from previous InSAR studies, 10 cm for Landers^18^ and 6 cm for Hector Mine^19^, is below the noise level and thus does not significantly distort our results.

### Spectral analysis of slip distributions

The slip distributions of both earthquakes exhibit along-fault variability at multiple length scales, with amplitudes exceeding measurement uncertainty. To understand how the displacement varies as a function of wavelength, we computed the power spectral density of the slip distributions using the multi-taper method (Fig. 2c,d)^20^. The spectral analysis reveals that the slip amplitude follows a consistent power-law decay over nearly three orders of magnitude in wavelength, showing slip is scale-invariant and fractal with no characteristic length scales dominating the frequency domain^21^,^22^. We find that the upper limit to the power-law behavior is the total length of the rupture; variability cannot exist at scales larger than that of the entire system. The lower limit is imposed by the image resolution; variability may well exist at smaller scales but is undetectable given the resolution of the data. To quantify the degree of roughness of fault slip, we estimated the fractal dimension (*D*) of both slip profiles from the power spectrum using the following relation, *D* = (5 − *β*)/2, where *β* is the slope in the power spectrum^22^, estimated using a linear least-squares regression past the corner frequency. The fractal dimension (*D*) characterizes the degree of slip roughness, for instance a value of 1 is the dimension of a line and 2 a surface, where higher fractional values denote an object that is more complex, deviating away from a smooth, straight line and attempting to fill a surface. From the slope in the power spectrum (Fig. 2c,d) we found for the Landers slip distribution *D* = 1.72 ± 0.02 (1σ), and for Hector Mine *D* = 1.62 ± 0.03 (1σ), indicating both are self-affine^22^, with Landers exhibiting a higher degree of variability. We obtained error estimates on the fractal dimension for both earthquakes using a Monte Carlo approach that simulates 10,000 possible slip distributions given the error in the displacement measurement (see Methods and Supplementary Figs S8 and 9). From these error estimates a T-test demonstrates that the Landers slip distribution is statistically rougher than that of Hector Mine (p-value of ≪0.001).

To illustrate the scale-invariant nature of slip, we isolate its amplitude at specific frequencies using narrow band-pass filters (Fig. 2e,f)^23^. This reveals that the slip variation is organized hierarchically, wherein the amplitude of slip variation decreases with wavelength. The longest wavelengths, near the fundamental mode, could be characterized as smooth elliptical or triangular distributions as in previous lower-resolution studies^1^,^8^ (also see Supplementary Fig. S1 for comparison of our displacement measurements with coarser results using a 10 m SPOT satellite data that illustrates this). It is important to note that the amplitude of short-wavelength variability does not imply unrealistically large along-strike strains; we observe strains of 7 × 10^−4^ for Landers and 4.5 × 10^−4^ for Hector Mine, values that are lower than the irreversible strain limit of the wall-rock (1 × 10^−3^)^24^, and similar to those observed from field and geodetic studies of other earthquake ruptures^5^,^6^,^25^.

### Analysis of fault system and relation to slip distribution

**Figure 3:**
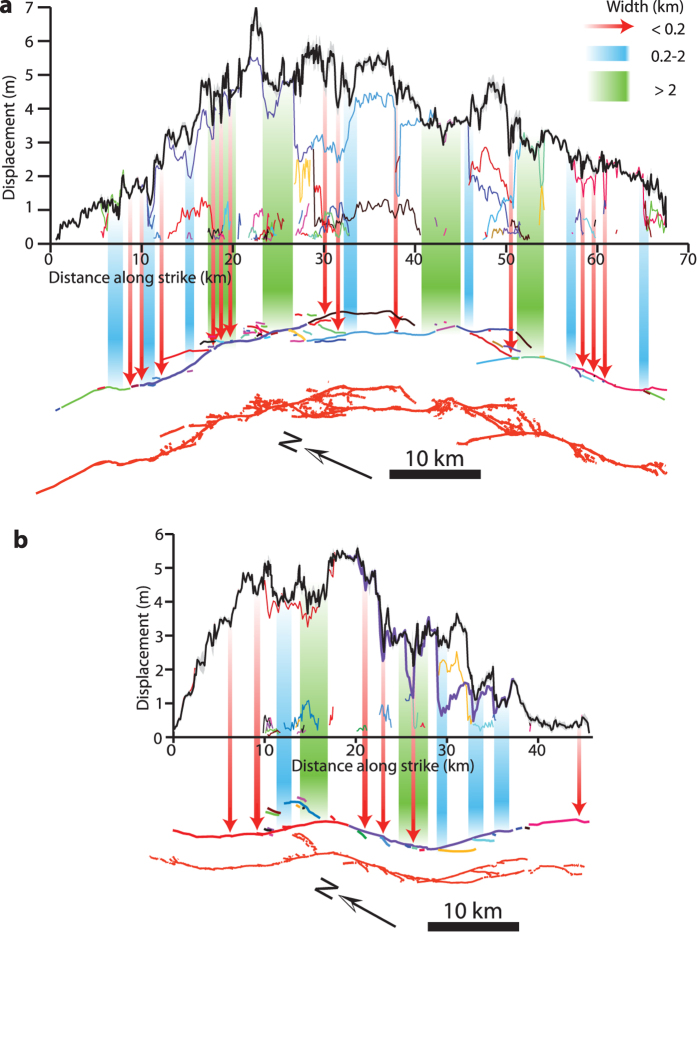
Correlation of slip variability (black line) to surface trace complexity. Slip profiles for (**a),** Landers and (**b),** Hector Mine (each plotted to same scale), where colored lines indicate individual fault slip profiles. Below slip profiles we plot the fault traces mapped in our correlation results (where colored traces correspond to individual colored slip profiles above), and the fault traces mapped in the field immediately following the earthquakes (red lines)^28^,^29^. Areas of long-wavelength slip (green, >2 km along-strike distance) correlate to large-scale jogs and bends in the fault trace, while intermediate-wavelength (blue, 0.2–2 km along-strike distance) and short-wavelength (red, <0.2 km along-strike distance) variability tends to correlate with progressively smaller-scale geometrical features. The >2 km category of slip variation is chosen so as to relate macroscopic variations of fault slip to macroscopic areas of structural complexity along the surface rupture, similar to the approach of ref. 30 and ref. 31. The shortest range of slip variation is chosen as the shortest resolvable variation in fault slip that we can observe along the rupture, with the medium wavelength category (0.2–2 km) simply defined as the variation of slip found between the two end-members.

Fault systems are known to exhibit geometrical complexities at all observable length scales and it has been widely recognized that fault surfaces themselves can be treated as scale-invariant and fractal^22^,^26^. To quantify the relative difference of the geometrical complexity between the two ruptured fault systems, we applied a boxcounting method^22^ to the rupture traces, measuring fractal dimensions of 1.29 ± 0.02 and 1.15 ± 0.02 (2σ) for Landers and Hector Mine, respectively (Supplementary Figs S10 and S11). A higher fractal value for the Landers fault system indicates it is geometrically more complex than Hector Mine at all scales, which is expected given the rupture propagated through five distinct fault segments and two structurally complex dilatational stepovers, whereas the Hector Mine rupture involved only three relatively well-defined faults^27^,^28^,^29^ (see fault trace maps in Fig. 3 and Supplementary Fig. S10).

Seismogenic-scale fault segmentation (e.g., macroscopic bends and stepovers) is well-known to correlate spatially to long-wavelength (~20 km) co-seismic slip variation^9^,^30^,^31^. Fault systems, however, are known to be segmented at not just one length-scale, but at multiple scales^22^, as demonstrated above. Therefore to test whether this relationship holds to smaller wavelengths, we employed a similar methodology of ref. 30 (see their Fig. 9) and ref. 31 (see their Fig. 2). First, we created three categories of slip variability based on wavelength (*λ*): short (*λ* < 0.2 km), intermediate (0.2 km ≤ *λ* ≤ 2 km), and long (*λ* > 2 km), where the shortest wavelength category is defined by the shortest resolvable changes of slip we can observe, the longest category defined by macroscopic geometrical fault complexities that relate to macroscopic changes of slip and the middle category simply defined as the middle of these two endmembers. For these three categories, we highlighted regions of noticeable slip variation (changes in slip greater than 15 cm, the 2σ measurement uncertainty) on the mapped surface trace (Fig. 3). Although subjective, this analysis yields clear results for both events; the wavelength and amplitude of slip variability generally corresponds to the local scale of geometrical complexity. For example, long-wavelength, large-amplitude slip variability (green arrows in Fig. 3) corresponds to large-scale macroscopic geometrical features such as kilometer-scale fault bends or stepovers, whereas shorter-wavelength, smaller-amplitude fluctuations (red arrows) correspond to sites of smaller-scale fault complexities such as branches and kinks. These results also illustrate the hierarchical organization of slip; the most common variability is the shortest wavelength and the least common is the longest wavelength. We note the correlation of areas of slip variation to sites of geometrical fault complexity are remarkably similar to those observed in the geomorphic record (Fig. 2 of ref. 31). Combined with the box-counting analysis of the surface traces, these results demonstrate that a more geometrically complex fault structure produces a rougher slip distribution; the more complex Landers fault system produces a rougher slip distribution at all scales, with a higher fractal dimension than the geometrically simpler Hector Mine event.

## Discussion

Field studies of fractally rough fault surfaces have found that fault roughness evolves with increasing slip; older, more ‘mature’ faults tend to be smoother with a lower fractal dimension^32^,^33^,^34^. This is consistent with our observation that the fractal dimension of the Landers fault system (i.e., degree of geometrical fault complexity) is higher than that of Hector Mine. The Landers fault system is known to have a slightly lower cumulative displacement (~3.1–3.6 km) than Hector Mine (between 3.4 and 7.8 km, ref. 35), indicating it is less structurally mature. Thus, the fractal dimension of the co-seismic slip distribution may provide a proxy for fault maturity and perhaps vice-versa.

Both quasi-static^36^ and fully dynamic numerical simulations^37^,^38^ of earthquake rupture have shown that a fractally rough fault surface induces a fractal stress field causing a highly variable slip distribution, and that increasing the roughness of the fault surface leads to a rougher slip distribution (see Fig. 4 of ref. 36 and Fig. 2 of ref. 38). This therefore provides a possible physical mechanism for our interpretation that the difference in fractal dimension of the slip distributions (i.e., roughness) between the two earthquakes can be explained by the relative difference of the fractal dimension of the fault structures (an overall measure of fault system roughness). The smoother Hector Mine fault system, with a lower number of sites of geometrical complexities, likely produces a spatially smoother stress field, resulting in a smoother co-seismic slip distribution at all scales.

However, a direct comparison between our results and those from quasi-static or dynamic rupture simulations assessing fault roughness on slip is challenging, given that our study measures the slip distribution from a heterogeneous 2D fault array, whereas numerical simulations have so far (i) used only single fault segments of fractal roughness and (ii) have parameterized rupture at seismogenic depths that are not subject to the same near-surface conditions as our measurements (e.g., velocity strengthening friction regime). Similarly, it remains to be seen whether the surface measurements of fractal values persist to depth. Therefore, although it is tempting to draw direct comparisons between our empirical values and those derived from finite-fault source inversions^39^, the use of different input data, parameterization of the fault geometry, and assumptions about elastic structure used to determine slip at depth precludes such a comparison with our data. In particular, regularization and interpolation of the final modeled slip distribution necessarily alters any existing self-similarity properties of co-seismic slip.

High-resolution displacement measurements of the 1992 Landers and 1999 Hector Mine earthquakes indicate that the complex spatial variation of co-seismic slip is a real feature and has a simple, predictable underlying fractal structure related to the hierarchical geometrical organization of the fault system. We find that the spatial frequency content of the fault structure can be observed within the slip distribution, indicating rougher faults systems produce rougher co-seismic slip at all scales. Synthetic self-affine fractal representations of co-seismic slip can be easily generated to serve as complex, realistic, and powerful models useful for seismic hazard analysis and understanding of slip distributions of paleo-earthquakes that are typically constrained by spatially sparse data. Our study also provides a novel approach for analyzing surface slip distributions of future earthquakes using high-resolution data, one that can explain the full heterogeneity of slip with a simple linear fit to the data in the frequency domain. This framework helps resolve longstanding questions concerning co-seismic slip variability, and creates a more complete understanding of the relationship between slip and fault structure.

## Methods

### Subpixel image correlation of air photos

For the Landers and Hector Mine earthquakes we selected 31 and 21 pairs of stereo-pair (60% overlap), 1 m resolution, National Aerial Photography Program aerial photographs, respectively, with 8 × 8 km footprint (purchased from http://earthexplorer.usgs.gov/). We selected air photos acquired in 1989, 1994 and 2002, where air photos from the two earlier flight missions serve as the pre and post Landers data and the latter two serve as the pre and post Hector Mine data. We note air photos from the 1994 flight mission serve as both the pre-Hector Mine and post-Landers dataset, giving rise to deformation maps for the two earthquakes derived from the same data acquired from the exact same optical sensor and flight mission, which helps minimize differences in data quality and accuracy. To produce correlation maps that accurately constrain the ground deformation pattern, the input aerial photographs must be precisely orthorectified and co-registered before correlation. The COSI-Corr program (http://www.tectonics.caltech.edu/slip_history/spot_coseis/download_software.html) allows for accurate orthorectification of images by taking into account the topography using a digital elevation model (DEM), the internal camera geometry using a camera calibration report (https://calval.cr.usgs.gov/calval_osl/calibration_reports/) to correct for optical distortions and the exterior orientation determined from ground control points (GCPs)^17^. To account for topographic distortion of the images, for both the Landers and Hector Mine events, we used the same 2012, 10 m National Elevation Dataset DEM that covers both ruptures, acquired from the USGS (http://ned.usgs.gov/). To georeference the post-event aerial photos, we used a 2005, 10 m, SPOT 5 image as the reference orthoimage for Landers and a 2000, 10 m, SPOT 4 image for Hector Mine. To co-register the pre and post-event photographs, we construct a relative mapping between image pairs using tie points that relates common features between pre and post-event image pairs. For co-registration GCPs are assumed to have experienced zero-ground movement, however, this assumption is violated due to long-wavelength ground deformation, to correct for this we used a correlation result from a pair of 10 m, SPOT 2 images for Landers and SPOT 4 images for Hector Mine^17^ (Supplementary Fig. S1), which provides independent constraint on ground motion. Topographic artifacts in the correlation result caused by use of only a single DEM to orthorectify both the pre and post-event photos are corrected for following the procedure of ref. 17. Once the pre and post-event air photos are orthorectified, COSI-Corr then applies subpixel image correlation to pairs of selected orthoimages by using an iterative, unbiased processor that estimates the phase plane in the Fourier domain^15^. For image correlation we used a multiscale sliding window of initial size of 64 and final size of 32 pixels with a step of 6 pixels, resulting in a correlation map of 6 m pixel resolution.

### Measuring displacement

Displacement is measured using stacked profiles orientated perpendicular to the fault strike, with lengths of 1–3 km and stack widths of 138 m, where the width defines the discretization of independent measurement. The optimal stack width of 138 m (23 pixels) is determined from synthetic tests (Supplementary Fig. S3–7), that gives a noise level of 2σ = 0.12 m in the displacement measurement, a noise level and stack width in agreement with previous work^16^, that allows for suppression of noise while minimizing over-smoothing along-strike changes in surface slip. Surface displacement is estimated from these stacked profiles by manually fitting linear regressions to either side of the fault, which are extrapolated to the fault trace to define the total amplitude of the discontinuity in the vector field, giving the magnitude of the total shear accommodated across the entire width of deformation (as shown in inset Fig. 1a,c). Where multiple fault strands exist within a single profile we measure displacement from each fault independently and simply sum these to give the total displacement accommodated across the system of faults.

### Synthetic tests

Estimating the magnitude of fault offset from the stacked profiles involves subjectively interpreting and fitting linear regression to the deformation signal, which can potentially lead to measurement bias and a possible component of artificial along-fault slip variation. To quantify the measurement precision and any possible bias that may arise in subjectively estimating displacement, as well as bias imposed by geometrical image distortions (such as scanning, thermo-mechanical warping or radial distortions) we employed a series of synthetic tests. In these tests we simulated synthetic fault ruptures through images with pre-determined constant along-strike fault displacement, where any deviation of the measured displacement from this known, spatially uniform synthetic value, directly quantifies the artificial amount of variation of displacement that arises from the subjective nature of estimating displacement, noise or artifacts within the correlation maps. These tests use the exact images and measurement process (i.e., correlation window parameters and stack profile dimensions) as that used to measure displacement from the real earthquake rupture, therefore allowing us to incorporate and asses the same noise, artifacts and measurement errors that would affect our real results. We note that we use MATLAB’s random number generator to produce the synthetic constant displacement so that the true value is not known when measurements of the displacement are estimated from the synthetic rupture. The true value is recorded, but not revealed to the user until after the measurements are complete, therefore avoiding any contamination from subjective bias. From these tests we derived an empirical error distribution (2σ = 12 cm) for displacement, in agreement with previous work^16^ (see Supplementary Figs S3–7 and Supplementary Tables S1–3). We note the synthetic tests reveal that the long-wavelength (>1 km) geometrical artifacts (e.g., radial distortions, scanning artifacts and thermo-mechanical warping) do not bias our measurement of displacement, as they occur at a wavelength an order of magnitude greater than the deformation signal (occurring at a length-scale of 1–100 m), (see Supplementary Figs S3–7), in agreement with previous studies^16^,^17^.

### Estimating the error of the slip profile fractal dimension

To obtain error estimates on the fractal dimension of the slip distributions for both earthquakes we used a Monte Carlo approach, that allows us to explore a large range of possible slip distributions given the error in the measurements. We generated 10,000 possible slip distributions for each earthquake (see Supplementary Fig. S8) by randomly sampling the error of each displacement measurement point (1081 displacement points for Landers and 470 for Hector Mine). From each of the simulated 10,000 slip distributions, we measured the fractal dimension by estimating the slope in the power spectrum from a linear least-squares regression (Fig. 2c,d)^22^, giving a range of possible fractal dimension’s that follow a Gaussian distribution (1σ of ±0.02 and ±0.03 for Landers and Hector Mine, respectively (see Supplementary Fig. S9)). We note, this method of generating a population of possible slip distributions (and therefore a distribution of possible fractal dimensions) from modeling the error of the displacement measurements in the slip profile, rather than simply using the error of the regression co-efficients of the ‘mean’ displacement profile in the power spectrum (i.e., range of slopes in Fig. 2c,d), is more robust, as we incorporate the effect of uncertainty originating from the data itself, as opposed to the error of estimating the power spectrum of a single possible slip distribution. This approach yields a wider range of possible fractal dimensions of the slip distributions of each earthquake, and therefore gives a more conservative estimate of the difference of slip roughness between the two events.

### Bandpass Filter

In Fig. 2e,f, we filter the slip distribution of both the 1992 Landers and 1999 Hector Mine earthquakes using a Butterworth filter^23^. This allows us to isolate the amplitude of slip at specific ranges of frequencies to illustrate how the amplitude of slip diminishes with decreasing wavelength. The upper and lower cut-off wavelength values for each band are chosen considering (i) numerical stability and (ii) illustrative purposes to clearly demonstrate how the amplitude of slip variation decreases as a function of wavelength. For each of the 10 bands used to filter the slip distribution shown in Fig. 2d,e, we used the following lower and upper cut-off wavelengths respectively for both earthquakes, 0.16–0.20 km, 0.25–0.33 km, 0.33–0.50 km, 0.5–1.00 km, 1.00–1.43 km, 1.43–1.66 km, 1.66–3.33 km, 3.33–6.66 km, 6.6–20 km, >20 km.

## Additional Information

**How to cite this article**: Milliner, C. W. D. *et al*. Resolving Fine-Scale Heterogeneity of Co-seismic Slip and the Relation to Fault Structure. *Sci. Rep.* **6**, 27201; doi: 10.1038/srep27201 (2016).

## Supplementary Material

Supplementary Information

Supplementary Dataset 1

Supplementary Dataset 2
