# Supplementary material for: Resolving Fine-Scale Heterogeneity of Co-seismic Slip and the Relation to Fault Structure
Source: Sci Rep. 2016 Jun 3;6:27201. doi: 10.1038/srep27201 (PMC4891690; doi:10.1038/srep27201)
Supplement: Supplementary Information [file srep27201-s1.pdf]

Supplementary Online Materials for:

Resolving Fine-Scale Heterogeneity of Co-seismic Slip and the Relation to Fault Structure

Author list: C. Milliner[1*], C. Sammis[1], A.A. Allam[2], J. Dolan[1], J. Hollingsworth[3], S. Leprince[4], F. Ayoub[5]

Affiliations:

[1]Department of Earth Sciences, University of Southern California, Los Angeles, California 90089, USA

[2]Geology and Geophysics, The University of Utah, Salt Lake City, Utah 84112, USA

[3] Maison des Géosciences, Université Joseph Fourier, Grenoble, France [4] Navdy, 575 7th Street San Francisco, CA 94103, USA

[5]Division of Geological and Planetary Sciences, California Institute of Technology, Pasadena, California 91125, USA

*Correspondence to: milliner@usc.edu

Validation of displacement measurements using SPOT data

To validate our displacement measurements derived from the 1 m National Aerial Photography Program (NAPP) air photos we used an independent dataset that can also measure the deformation field and slip distributions of both the Landers and Hector Mine earthquakes. Our independent data consists of pre- and post-event 10 m SPOT imagery that cover both earthquakes in space and time, giving an independent constraint on the surface deformation field and slip distribution, which is produced from the same COSI-Corr procedure as that used for the air photos.

The Landers SPOT correlation (Supplementary Fig. S1 a,b) is produced from a pre-event, 1991, 10 m SPOT 2 image, and a post-event, 1993, 10 m SPOT 2 image. For Hector Mine we produced the correlation maps (Supplementary Fig. S1 d,e) from pre-event 1993, 10 m SPOT 4 imagery and post-event imagery from a 2000, 10 m SPOT 4 image. For both earthquakes we used a multi-scale correlation window of initial 64 pixels and final of 32 pixels with a step size of 8 pixels, similar correlation parameter as that used for the air photo data, and applied a non-local means filter to the correlation result to suppress noise. To measure the displacement we used stacked profiles of 2.5km width, spaced with the same distance along the surface rupture.

Comparing the slip distribution from the SPOT correlation to the air photos we find an excellent agreement at the first-order scale (Supplementary Fig. S1). The overall smoothness of the SPOT result is a function of a (i) coarser pixel resolution (10 m of SPOT data compared to 1 m air photos), (ii use of a non-local means filter to reduce noise in the correlation result and (iii) use of wide stacked profiles (2.5 km compared to 138 m in the air photos) with also the same coarse measurement discretization. Therefore the excellent agreement between these two

different geodetic datasets at the first-order scale, which is what the SPOT imagery can resolve, provides a robust validation of our displacement measurements.

Synthetic tests

To quantify the measurement precision and any possible bias in estimating displacement we employed synthetic tests. Specifically, we design these tests to capture and quantify how the estimation of displacement measurement is effected by (i) noise and geometrical artifacts within the correlation maps and (ii) the subjective manner when interpreting the deformation signal within the stacked profiles. To do this we create a synthetic rupture through the air photos with pre-determined displacement that is constant along the fault, which is then measured using stacked profiles in order to quantify how well we can recover the spatially uniform values. Thus when measured if any spatial variation of displacement does arise that deviates from the known constant value, this directly constrains the effect of noise, long-wavelength geometrical artifacts (such as scanning, thermo-mechanical warping or radial distortion) from the correlation maps and the subjective interpretation of estimating displacement.

To simulate a synthetic rupture on a 'fault' we simply shift one half of a post-event, aerial image by a pre-determined amount. To generate a displacement for the synthetic fault rupture without it being known to the user when measuring displacement we use MATLAB's random number generator, where the value is recorded and only revealed until after the measurements are complete, therefore allowing us to conduct measurements of displacement without knowing the true value and biasing the results. We correlate the artificially dextrally sheared image with a different non-disturbed pre-event image (Supplementary Fig. S2) using the same processing procedure in COSI-Corr that we use to produce our real dataset. We measure displacement using stacked profiles of 138 m width and lengths up to a maximum of 3 km. Where multiple fault

strands exist within a single profile we measure displacement from each fault independently and simply sum these to give the total displacement accommodated across the system of faults.As the images are aquired over areas that contain real tectonic deformation, which are areas we want to neglect from our tests (as this would contaminate our synthetic known ruptures), we simply used information from the real correlation result (Fig. 1 of the main text) to help avoid such areas. To shift image pixels by a non-integer amount, we applied a cubic interpolation to one side of the deformed image. In total we used five image pairs, one pair from the Landers (Supplementary Fig. 2 a and b) event and four pairs from the Hector Mine event (Supplementary Fig. 2 d, e, g and h), where each pair contains a pre and post-event image..

As the synthetic offset is determined to be constant along the entire fault length, any deviation of the measurements from the synthetic, known values gives a direct quantification of the measurement bias and precision, which reflects measurement subjectivity and variation in image quality and texture. Furthermore repeatedly measuring the displacement along-strike of our synthetic fault across different parts of the correlation image gives multiple independent measurements of displacement. We estimate measurement bias by simply subtracting the average displacement (from a large sample) that we measure from the correlation maps, to the true, known synthetic displacement value. To avoid biasing the measurements, the true, pre-determined synthetic fault offset values were chosen using a random number generator and were not revealed to the user until the measurements from the correlation maps were completed.

In total we performed three types of synthetic tests that utilized an array of faulting styles, widths and displacements expected in a real environment from imagery acquired over both the Landers and Hector Mine events, that are outlined in detail below (Supplementary Fig. S2).

Results

The first test involved imagery associated with the Landers event with the aim of understanding whether COSI-Corr's sub-pixel correlation behaves differently when evaluating fault displacement that exceeds a pixel dimension i.e., 'supra' pixel fault movement. To test whether COSI-Corr gives consistent results and does not artificially alter displacement if the fault movement exceeds a pixel dimension, we used one fault with supra-pixel movement of 2.5 m and a second with sub-pixel movement of 0.4 m displacement (where the image pixel resolution we use is 1 m). We used fault lengths of ~7 km that span the entire image and aquired 45 displacement measruements for each fault. We note this first test is similar to that conducted by ref. 40 found in the supplementary file, and we re-performed this test in order to establish consistentcy between the two studies.

The first synthetic test reveals an overall bias of 0.02 m with a precision of $\pm$ 0.04 m (1σ) for our displacement measurements (Supplementary Fig. S3). Specifically, both the sub- and supra-pixel displacement tests yield the same behavior with a 1σ measurement precision of $\pm$ 0.041 m and $\pm$ 0.038 m, respectively, in agreement with previous studies [1,2].

The second and third tests involved imagery used for the correlation of the 1999 Hector Mine event, again, from locations that contain no real tectnoic deformation. Specifically, the second test invovles performing additional displacement measurements, similar to the first test, in order to understand whether the measurement uncertainity is different from the correlation produced by air photos associated with the Hector Mine earthquake in comparison to air photos used for the Landers event (even though the post-Landers and pre-Hector Mine are derived from the same 1994, NAPP flight mission). For test 2 we implemented six faults of equal length and spacing but with different magnitudes of displacement, that were set constant along-strike (Supplementary Fig. S2 d,e,f). In total we collected 434 displacement measurements, finding an

overall bias of 0.01 m and precision (1σ) of ± 0.06 m (Supplementary Fig.S 4c). Importantly, we found no discernable diffference of the measurement precision or accuracy derived from the Landers air photos (test 1, with a bias of 0.02 m and 1σ of ± 0.04 m) or the Hector Mine imagery (see Table S1 and S2 and Supplementary Fig. S3 and S4).

The third test involved changing the width of deformation to understand whether a wider fault zone causes the measurement estimation to be more amibigous and therefore more difficult to interpret and adequatley extract the true displacement. To simulate 'faults' of various widths we simply varied the number of multiple parallel fault strands within a synthetic 'fault zone'. We simulated six different main 'fault zones' with widths of 1, 25, 50, 100, 125 and 150 m, that are of constant width and displacment along-strike. We simulate widths that are similar to those observed in the real correlation results and that observed from [1,3,4]. From this third test we collected 435 displacement measurements and found an overall measurement bias of 0.02 m and precision of 1σ ± 0.06 m (Supplementary Fig. S5). Importantly, we found the displacement bias and precision is not significantly affected by the width of deformation (Supplementary Fig. S6). The similar level of bias and precision found in test 3 to that of test 1 and 2, again indicates the width of deformation has an unnoticeable effect on the estimation of displacement in the stacked profiles.

For all three tests we found the spectrum of these results is close to but does not follow white noise (Supplementary Fig. S7), in agreement with a previous and similar analysis [2] and importantly, nor do they have the same slope in the power spectrum as the real displacement measurements reported in the main text. The slope of the slip spectra for tests 2 and 3 are 0.31 (corresponding to a fractal dimension [D] = 2.34) and 0.47 (D = 2.26), respectively (Supplementary Fig. S7 b and d). Furthermore, we note that both the slope and the power of the

synthetic slip in the spectra are significantly diferrent (and less in power) than that found in the real results presented in the main text, again indicating variation of slip induced by measurement subjectivity and noise within the images, is insufficient in amplitude across a broad range of frequencies to cause the observed amplitude of slip variaiton that is observed in our real correlation maps (Fig. 1).

From all three synthetic tests, we found a consistent value for the measurement bias and precision of displacement when estimated from the stacked profiles. From a total of 959 displacement measurements from the three synthetic tests, we found an overall measurement bias of 0.02 m and precision (1σ) of ± 0.06 m with no observable systematic variation as a function of the air photos used (Landers or Hector Mine flight missions), fault width or magnitude of displacement. Using these results we derived an empirical error distribution for the displacement measurements (Supplementary Fig. S7c), which we propagate through the pre-existing error in COSI-Corr, that forms the basis of the measurement uncertainty found in the main text. The small precision and measurement bias is reflective of the robustness of the correlation procedure, the quality of the aerial images, and the use of profile stacking to suppress noise. These tests also confirm the width of our stacked profiles is appropriate, a width that reduces noise to a low level of uncertainty (1σ = 0.06 m), the same as that observed from ref. 17, that found using the same Landers images used here, this value of uncertainty is the base level of noise in the correlation result between the before and after images. Thus, these results and their agreement with previous work indicate a stack profile of 138 m width is sufficient to reduce noise to its floor level, but not unnecessarily wide to ‘over-smooth’ displacment along the surface rupture. We note that Figs. S3, S4, S5 and S7 show no evidence of long-wavelength artifacts (such as scanning, thermo-mechanical warping or radial distortions) biasing the displacement measurements, as we found

the mean measured displacement values were consistent with the true synthetic value with no systematic offset. We note the synthetic rupture occurs in areas where scanning and thermo-mechanical distortions are present in the images (as can be observed in the correlation result of Supplementary Fig. S2c) and we find these geometric distortions do not influence or offset the measured displacement from the true value. We note our results showing the long-wavelength artifacts do not bias our measurement of displacment, a signal which occurs over <100 m length scales is in agreement with previous studies[16,17,40]."

Estimating the statistical difference between the fractal dimensions of the 1992 Landers and 1999 Hector Mine slip distributions.

From each of the simulated 10,000 slip distributions for the Landers and Hector Mine earthquakes, derived using the Monte Carlo approach (see Methods), the fractal dimension was estimated from the slope in the power spectrum beyond the corner frequency from a linear regression calculated using a least-squares approach (Fig. 2b and e) [5]. The corner frequency (red vertical lines in Fig. 2 c and d) in both cases are determined by the rupture length which limits the long possible wavelength (i.e. the fundamental mode) of slip that can occur along the rupture.

For a total of 20,000 slip distributions (10,000 for each earthquake), we found that the estimate of the fractal dimension follows a Gaussian distribution with a 1σ of $\pm$ 0.02 and $\pm$ 0.03 for Landers and Hector Mine, respectively (Supplementary Fig. S9). Using a T-test we compared the statistical significance of the difference between the fractal dimension of the Landers (1.72 $\pm$ 0.02) and Hector Mine slip distribution (1.62 $\pm$ 0.03) using the values obtained from the Monte Carlo simulations. We found the T-test rejects the null hypothesis that the fractal dimension of the Landers and Hector Mine slip distributions are drawn from the same distribution, with a p-value of 0.0014, well below our confidence level of 5%.

Boxcounting of the Landers and Hector Mine fault systems

Fault systems and fractures have been shown to follow fractal distributions and previous studies have characterized the fractal properties of faults using boxcounting methods[41,42]. Here we used a boxcounting method to estimate D in order to quantify which fault system, Landers or Hector Mine is more complex and whether the geometrical complexities that compose these faults systems can be considered scale invariant and fractal structures. The boxcounting method allows one to draw log-log plots of the number of boxes required to cover the object (N_r), in this case the mapped fault traces, against the size of the box (r). The slope of the log-log plot provides an estimation of D, that is between 1 and 2 in this case.

To be considered a fractal object the boxcounting curve needs to follow a straight line in log space, defined by the following relation: $\log(N_r) = a + D * \log(1/r)$ [5]. To estimate the fractal dimension we used boxes of decreasing size and count the number of them that cover the mapped surface fault traces (Supplementary Fig. S10). We use boxes of sufficient size that adequately covers a range of spatial scales similar to that of the distance between individual fault strands.

The fault traces were acquired from the USGS (http://earthquake.usgs.gov/hazards/qfaults/google.php), and were produced by careful mapping immediately after both earthquakes [3,8]. Therefore importantly the fault traces analyzed here represent the faulting directly involved in the rupture and are not inactive and therefore irrelevant structures.

To minimize any possible distortions or avoid introducing any potential artifacts, we take a single high resolution image that includes both the fault systems of Hector Mine and Landers,

as they are fortuitously only 20 km apart. Therefore both fault traces are projected in the same map projection system (UTM WGS-84, Zone 11N) causing minimal distortion to the geometry of the fault traces and imaged at the same resolution (700 dots per inch) and scale (1:400,000).We then split the image into two in order to appropriately separate the Landers and Hector Mine fault systems and then apply the boxcounting analysis separately to each image (Supplementary Fig. S10). Thus from this procedure we can be confident any difference in the estimation of the fractal D from the boxcounting method is primarily a reflection of fault geometrical complexity rather than a reflection of any spurious artifacts.

Results

We analyze the boxcounting curve over more than 2 orders of magnitude (10^2 –10^4 pixels). From a linear regression to the boxcounting curve we find a fractal dimension of 1.29 $\pm$ 0.01 with R^2 = 0.98 for the Landers fault system and 1.15 $\pm$ 0.01 with R^2 = 0.99 for the Hector Mine rupture, which is seen in Supplementary Fig. S11 as a steeper curve. Thus, the boxcounting method clearly indicates the Landers surface rupture has a more complex geometry than the Hector Mine rupture, which can also be easily validated with a simple visual comparison of the two surface fault traces of the two earthquakes (Supplementary Fig. S10). We also tested whether the initial box size affects the estimation of the fractal dimension by rotating the faults (Supplementary Fig. S10). We find an insignificant difference when varying the initial box size (by rotating the images of the fault traces) with a difference of 0.0023 and 0.0062 in the fractal dimension for the Hector Mine and Landers surface fault traces respectively.

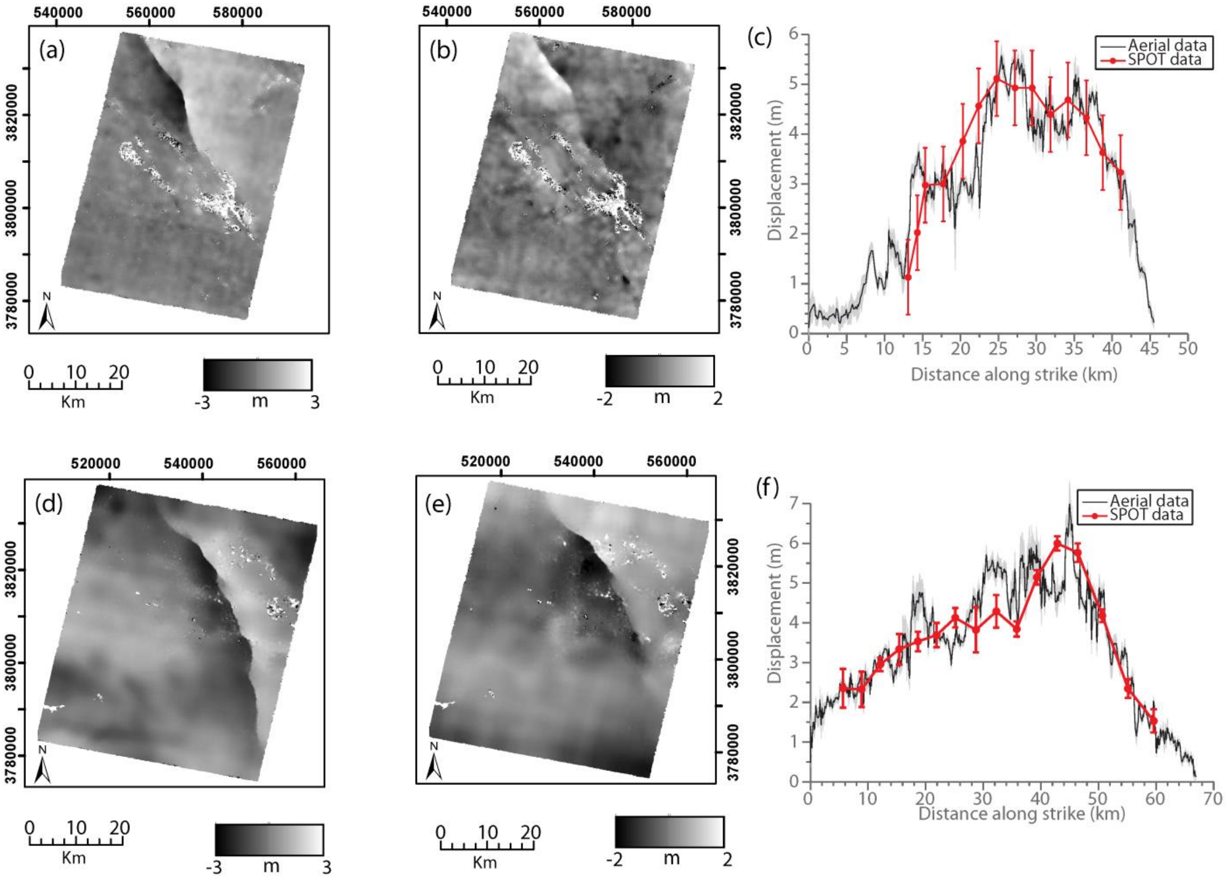

Supplementary Figure S1. Correlation results and slip profiles of Hector Mine and Landers event using SPOT satellite imagery. a) SPOT correlation result showing north-south motion of ground surface of Hector Mine earthquake b) SPOT correlation result of Hector Mine earthquake in east-west direction. c) Slip profile of Hector Mine event, black showing data measured from air photos presented in main text and red line showing displacement measured from correlation maps seen in a) and b). d) SPOT correlation result showing north-south motion of ground surface of Landers earthquake e) SPOT correlation result of Landers earthquake in east-west direction. f) Slip profile of Landers event, black showing data measured from air photos presented in main text and red line showing displacement measured from correlation maps seen in a) and b). The displacement maps were computed using COSI-Corr and plotted within ENVI 4.8

(http://www.exelisvis.com/ProductsServices/ENVIProducts/ENVI.aspx) and Arcmap 10.1 (http://www.esri.com/software/arcgis/arcgis-for-desktop).

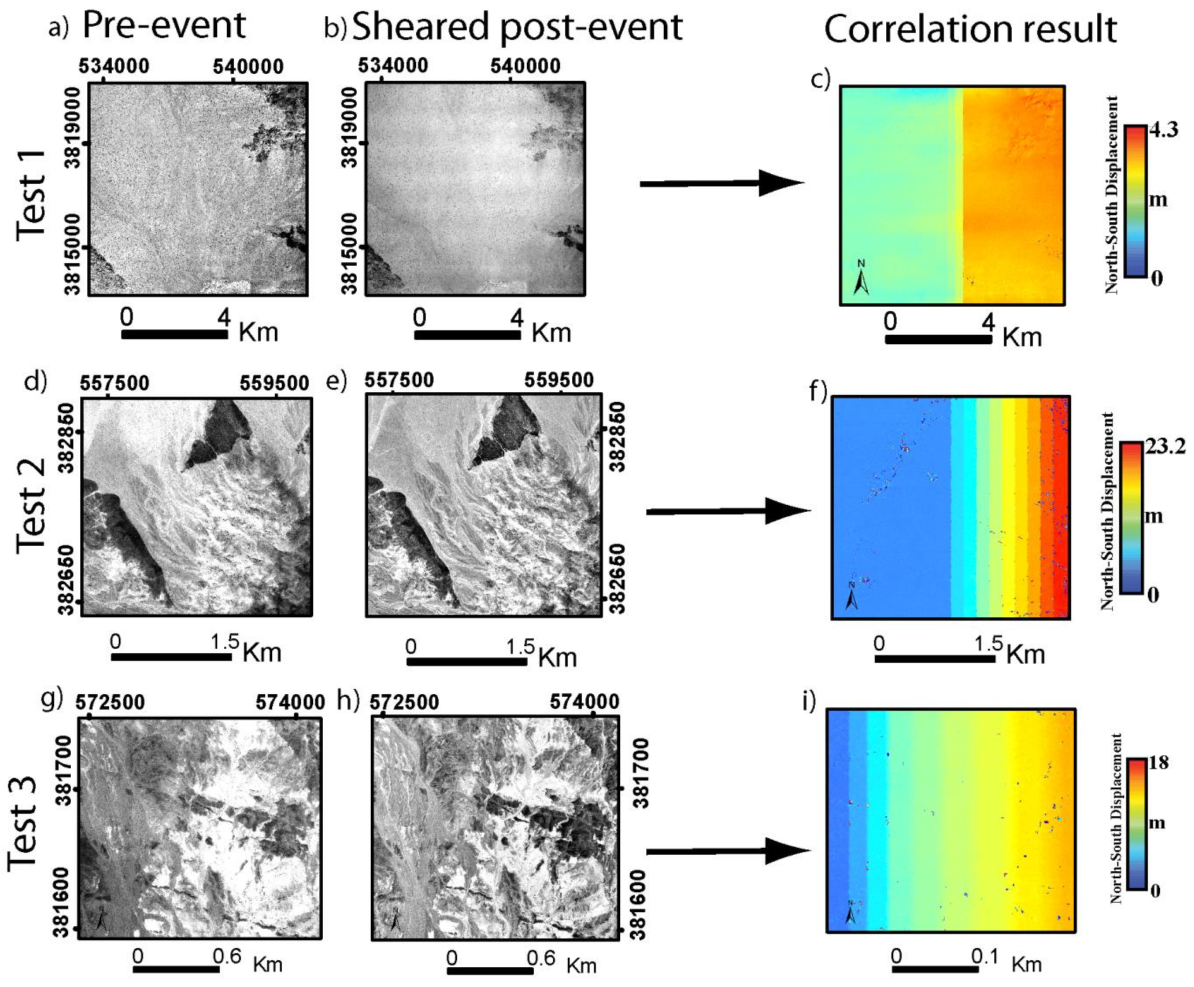

Supplementary Figure S2. Illustrates a subset of the images used for the three synthetic tests to empirically determine the measurement uncertainty. Left column of figure S2 (a, d and g) shows the pre-event images, middle column of S2 (b, e and h) show post imagery that was synthetically sheared to produce faults of various widths and displacement (both constant along-strike) and right column shows (c, f and i) correlation result where displacement measurements were taken from faults running north-south through images (seen as vertical lines juxtaposing different

amounts of ground motion denoted by different colors). Test 1 involves two faults one of supra-pixel displacement (2.5 m) and sub-pixel displacement (0.4 m). Test 2 involved faults of different displacement but constant width and test 3 faults of different displacement and different widths. We note in each of tests 2 and 3 we used two pairs of air photos (two pre- and two post-event) allowing us to acquire more measurements, giving a total of four pairs of air photos used for the two tests, where Supplementary Fig. S2 d,e,g and h, show subsets of these air photos. The displacement maps were computed using COSI-Corr and plotted within ENVI 4.8 (http://www.exelisvis.com/ProductsServices/ENVIProducts/ENVI.aspx) and Arcmap 10.1 (http://www.esri.com/software/arcgis/arcgis-for-desktop). Air photo data compiled by the U.S. Geological Survey (http://www.usgs.gov).

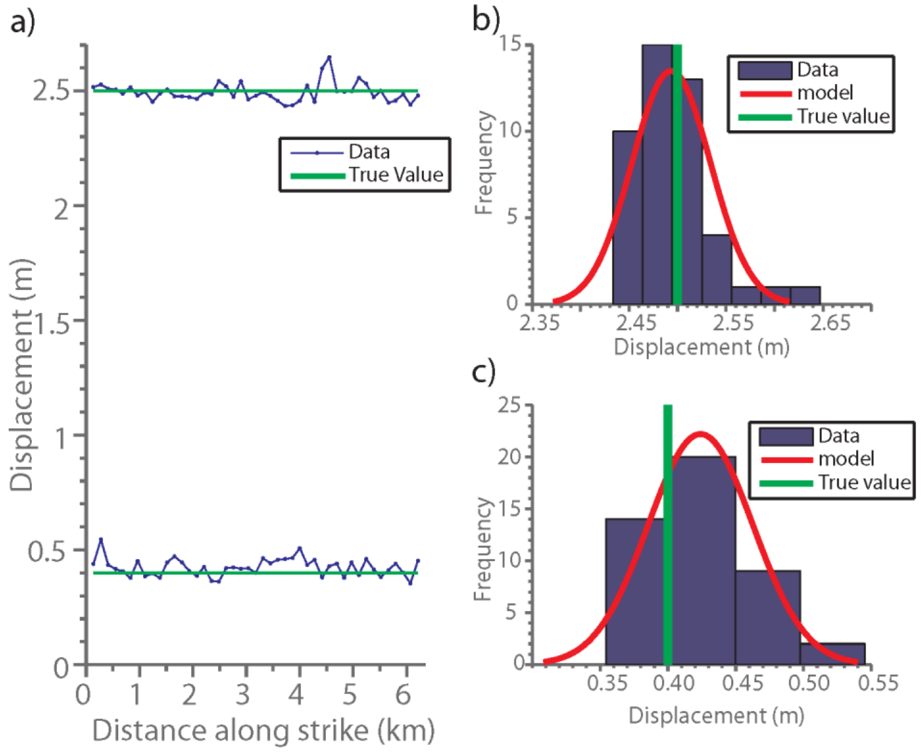

Supplementary Figure S3. Measurement results from synthetic test 1. a) Slip profiles of displacement measurements taken from two faults with 2.5 (supra-pixel displacement) and 0.4 m

(sub-pixel displacement). Green lines show the true, synthetic displacment value and blue dots and line show the displacment measurements aquired from the stacked profiles from the correlation maps (Fig S2c). b) Shows the histogram of measurements (n = 45, shown as blue bars) from the supra-pixel displacement test that follow a Gaussian distribution (red line) with a true, synthetic value of 2.5 m that is labeled by a green vertical line. The offset of the true synthetic value from the mean measured values is 0.01 m, measurements from the tests yield a mean of 2.49 m, a 1σ of $\pm$ 0.04 m and median of 2.49 m. c) Shows the histogram of measurements (n = 45) from the sub-pixel displacement test (blue bars) that follow a Gaussian distribution (red line) with a true, synthetic value of 0.4 m that is labeled by a green vertical line. The offset of the true synthetic value from the mean measured values is 0.02 m, measurements yield a mean of 0.42 m, a 1σ of $\pm$ 0.038 m and median of 0.42 m.

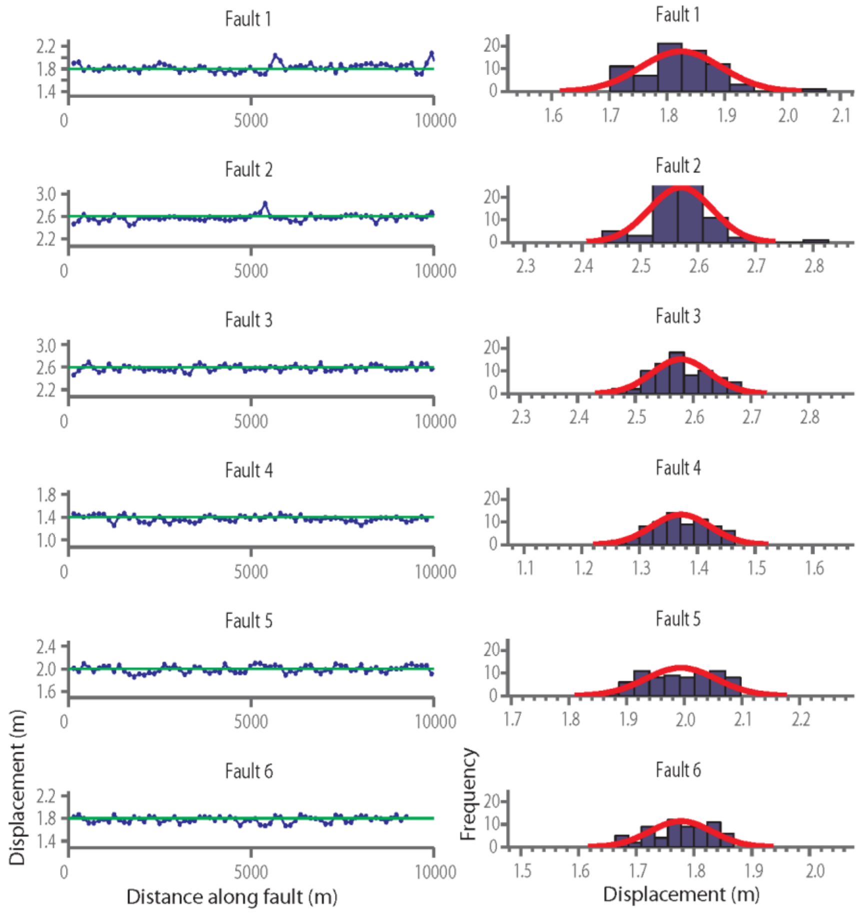
Fault 1
Fault 2
Fault 3
Fault 4
Fault 5
Fault 6
Displacement (m)
Distance along fault (m)
0
5000
10000
Frequency
Displacement (m)

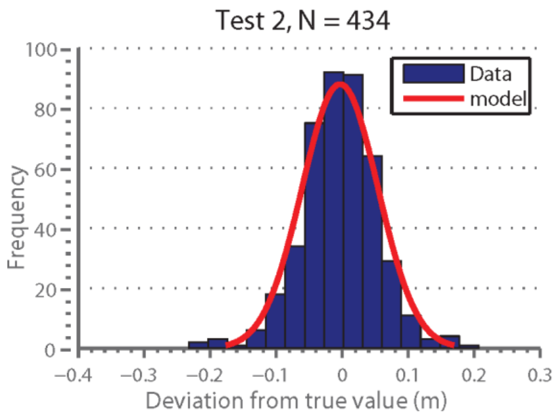
Test 2, N = 434
Data
model
Frequency
Deviation from true value (m)

Supplementary Figure S4. (top) Displacment measurements from test 2 (Supplementary Fig. S2f), left side of figure shows slip profile of each fault, right side of figure shows the distribution of data where each row represents the displacement measurements from each of the six synthetic ‘faults’. (bottom) shows the overall distribution of all measurements from test 2 (n = 434), which has been subtracted from its respective true, known synthetic value. We found an overall mean (i.e., bias) of -0.0034 m, 1σ of $\pm$ 0.06 m and median of -0.0029. See Table S2 for statistics for each fault.

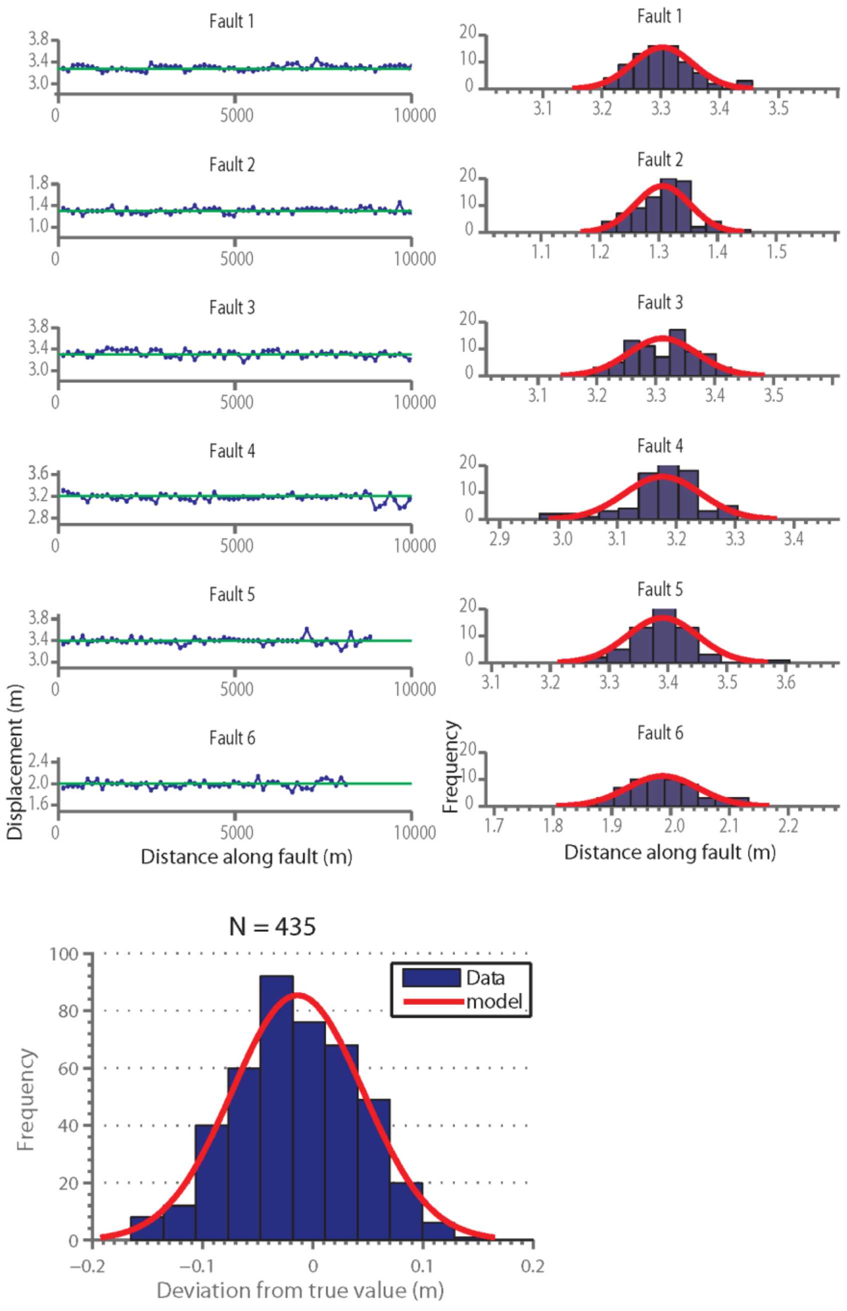

Supplementary Figure S5. (top) Displacment measurements from test 3 (see Supplementary Fig. S2i), left side of figure shows slip profile of each fault, right side of figure shows the distribution of data where each row represents the displacement measurements from each of the six synthetic ‘faults’. (bottom) shows the overall distribution of measurements from test 3 (n =

435), which has been subtracted from its respective true, known synthetic value. We found an overall mean (i.e., bias) of -0.01 m, 1σ of ± 0.06 m and median of -0.01. See Table S3 for statistics for each fault.

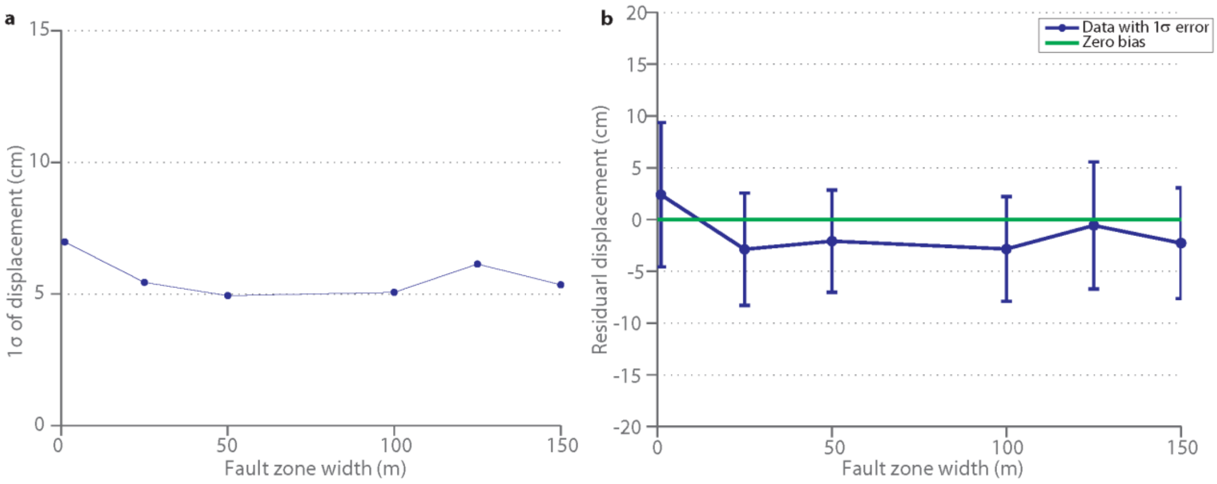

Supplementary Figure S6. a) The variation of displacement measurements (1σ) plotted as a function of fault zone width. We see no change in the variability of displacement measurement with the width of the fault zone. That is, a wider fault zone does not make the interpretation of the deformation signal significantly more ambigious and therefore faults of different widths do not artificially cause an increase in the variance of the displacement measurements. b) Displacement measurements from test 3 plotted as function of fault zone width. We find there is no systematic change of the displacement bias (difference from true value) with width of the fault zone. That is, a wider fault zone does not make it more difficult to accuratley estimate the displacment measurement.

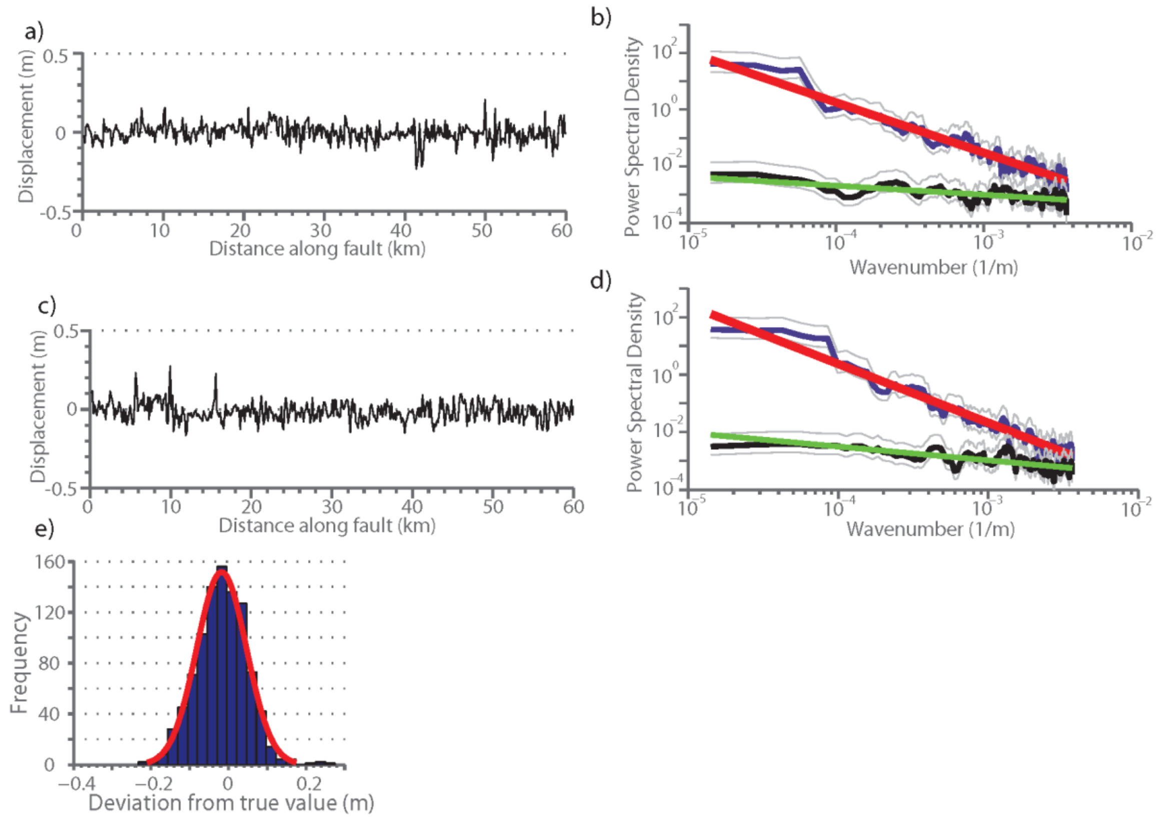

Supplementary Figure S7. a) and c) show all displacement measurements from tests 2 and 3, respectively. Power spectrum of synthetic tests 2 and 3 plotted in b) and d) respectively as black lines (green lines show linear fit), with the real measurements of Landers and Hector Mine fault slip plotted as blue lines (red line showing linear fit) in b) and d), respectively. The figure clearly illustrates that the spectra of the synthetic tests have a different slope and significanlty lower amplitude than the true measurements, indicating the variation of slip observed in the actual earthquakes is primarily real and reflecting the rupture process as opposed to meaurement uncertainty. e) Shows the distribution of all displacment measurements aquired from tests 1, 2 and 3 (n = 959), subtracted from their true known value. We find mean (i.e., bias) of 0.02 m, 1σ of ± 0.06 m and median of 0.02 m. We use this distribution as our empirical error distribution that is used for the measurement uncertainty presented in the main text.

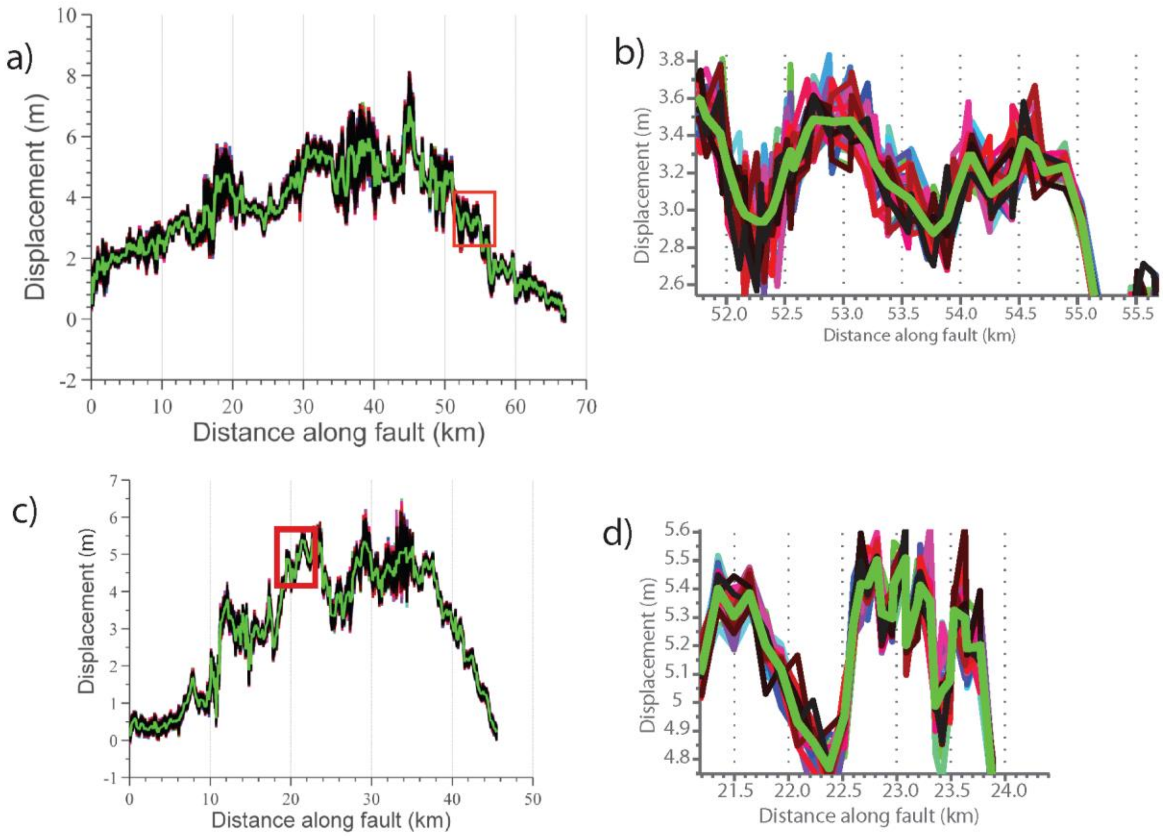

Supplementary Figure S8. Simulated slip distributions for the Landers (top) and Hector Mine earthquakes (bottom) determined by a Monte Carlo method a) 10,000 possible slip distributions for the Landers earthquake given the error of the measurements, thick green line shows the mean slip distribution that is directly measured from the stacked profiles. b) Zoom-in of a 3.5 kilometer area denoted as a red rectangle in a), to illustrate how the Monte Carlo simulation randomly samples the error to produce different possible slip distributions. Note for illustrative purposes in b) and d) we only plot 30 possible slip distributions in order to adequately see each individual simulations. c) 10,000 possible slip distributions for the Hector Mine earthquake, given the error of the measurements. d) Zoom-in of a 2.5 kilometer area denoted as red rectangle in c), to illustrate how the Monte Carlo simulation randomly samples the error to produce different possible slip distributions.

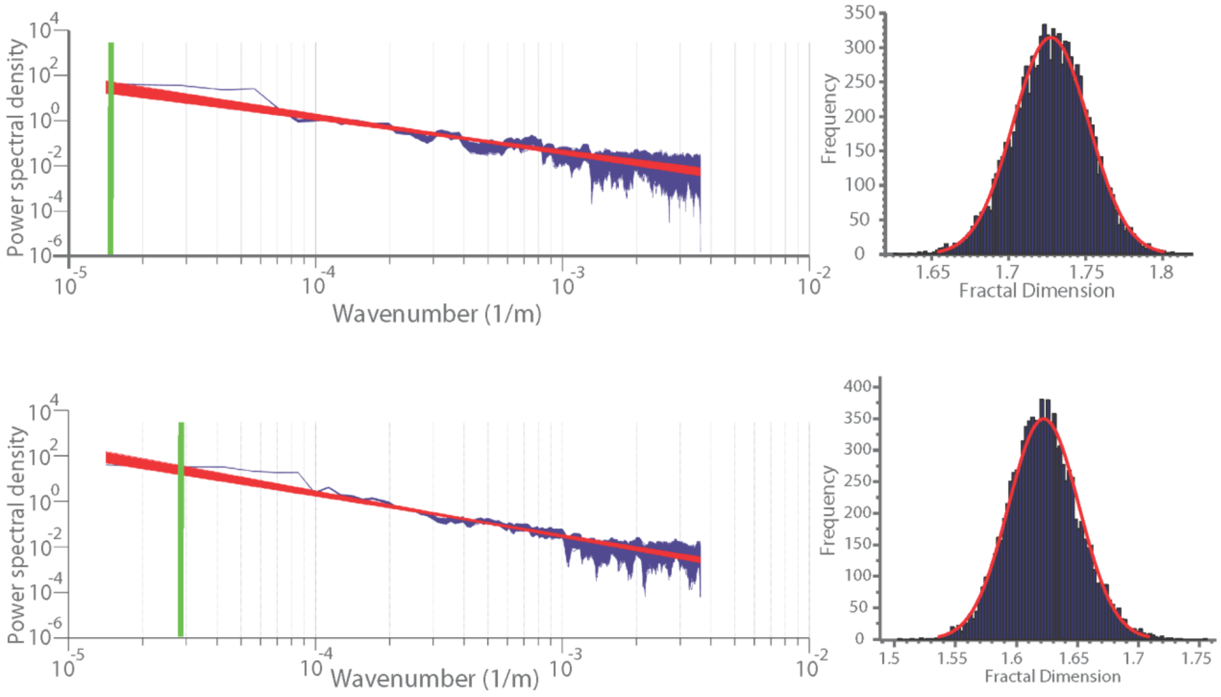

Supplementary Figure S9. a) Power spectrum for 10,000 possible slip distributions of the Landers earthquake produced from randomly sampling the error using Monte Carlo simulations. Red lines are 10,000 linear regressions fit to each of the 10,000 independently estimated spectra of the simulated, possible the slip distributions. Green vertical line shows the location of the corner frequency which delineates the extent of the fitting of the linear regression and corresponds to the length of surface rupture (67 km). b) Histogram of 10,000 possible fractal dimensions for the Landers slip distribution, which follow a Gaussian distribution (red line) with mean of 1.72 and 1σ of $\pm$ 0.02. c) Power spectrum for 10,000 possible slip distributions of the Hector Mine earthquake produced from randomly sampling the error using Monte Carlo simulations. Red lines are 10,000 linear regressions fit to each of the 10,000 independently estimated spectra of the simulated, possible the slip distributions. Green vertical line shows the location of the corner frequency which delineates the extent of the fitting of the linear regression and corresponds to the length of surface rupture (38 km). d) Histogram of 10,000 possible fractal

dimensions for the Hector Mine slip distribution, which follow a Gaussian distribution (red line) with mean of 1.62 and 1σ of ± 0.03.

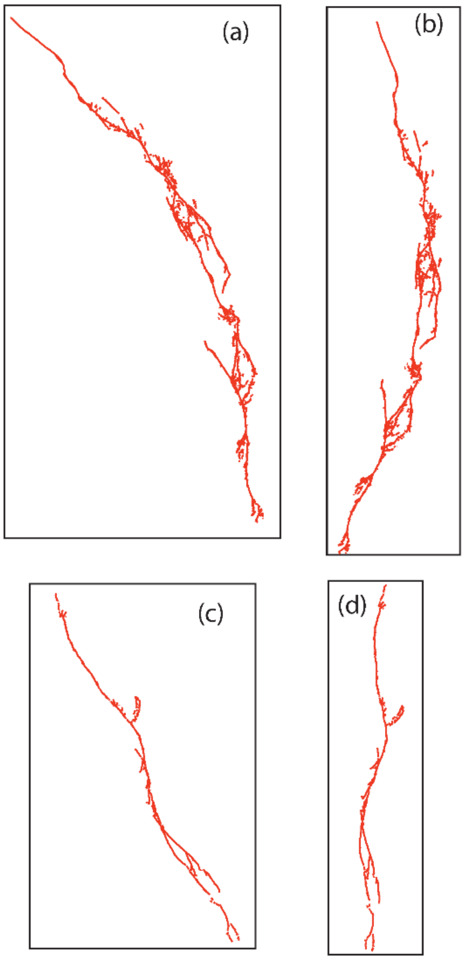

Supplementary Figure S10. (top) Map view of the surface fault traces of the Landers [8] and bottom the Hector Mine rupture [3] mapped in the field, with the left column showing the fault traces in their north-orientated position and right column showing the fault systems rotated to minimize the amount of image space it occupies. We show here the actual images used in the boxcounting procedure and therefore do not show scale or north arrow, however, all four images are at the same 1:400,000 scale. We find no significant difference in the fractal dimension between which orientations are assumed for the fault traces.

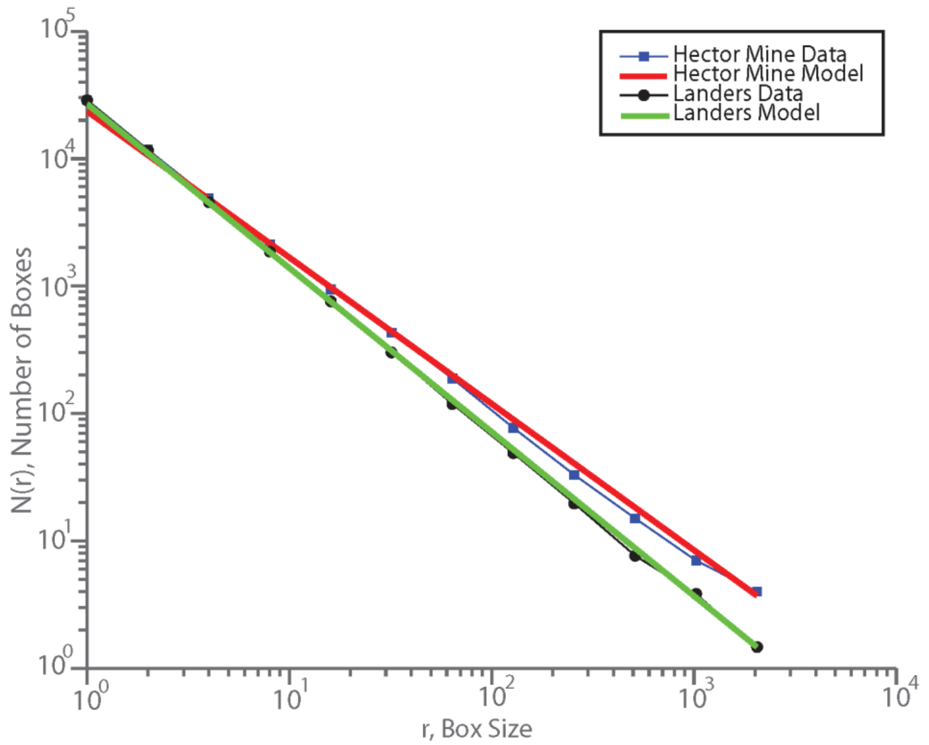

Supplementary Figure S11. a) Log-log plot of number of boxes (N_r) versus size of boxes (r) from the boxcounting of the surface fault traces. The blue line and solid circles shows the boxcounting result for the surface fault traces of the Hector Mine event, with the red line showing the fit giving a D = -1.15 $\pm$ 0.01. The black line and solid circles shows the boxcounting result for the surface fault traces of the Landers earthquake, with the green line showing the linear regression giving a D = -1.29 $\pm$ 0.01. We note for the purpose of illustrating the difference in slope between the two fault systems we have shifted the Landers boxcount curve vertically down the y-axis by a factor of 3.4 in log space.

Supplementary Table S1 Statistics of measurements from synthetic test 1 for two faults of sub-pixel displacement (row 2) and supra-pixel displacement (row 3).

**True fault displacement (m)**	**Mean measured displacement**	**1σ of measured displacement (m)**	**Bias** (i.e. mean difference of measured	**Number of measurements**

	**(m)**		value to known true value) (m)	**on fault**
0.40	0.42	0.04	0.02	47
2.50	2.49	0.04	-0.01	47

Supplementary Table S2. Statistics of measurements of test 2 with six faults of different displacements, with constant widths.

**True fault displacement (m)**	**Mean measured displacement (m)**	**1σ of displacement measurement (m)**	**Bias** (i.e. mean difference of measured value to known true value) (m)	**Number of measurements on fault**
3.30	3.30	0.05	0.00309	79
1.30	1.29	0.05	0.00672	79
3.30	3.31	0.06	0.01	77
3.20	3.18	0.06	-0.02	76
3.40	3.39	0.06	-0.01	64
2.00	1.99	0.06	-0.01	59

Supplementary Table S3. Statistics of measurements of test 3, from siz faults with different displacements and widths of deformation.

Fault width	**True fault displacement (m)**	**Mean measured displacement (m)**	**1σ of displacement measurement (m)**	**Bias** (i.e. mean difference of measured value to known true value) (m)	**Number of measurements on fault**
0	1.80	1.82	0.07	0.02	74
25	2.60	2.57	0.05	-0.03	76
50	2.60	2.58	0.05	-0.02	75
100	1.40	1.37	0.05	-0.03	71
125	2.00	1.99	0.06	-0.01	74
150	1.80	1.78	0.05	-0.02	67

Supplementary Table S4: Due to the large size, this table is part of the Data repository. Measurements of displacement for the 1992 M_w 7.3 Landers earthquake. Column 1 and 2 contains gives the location of the displacement measurements from the stacked profiles in Latitude and Longitude, column 3 details the displacement and column 4 the 1σ uncertainty of the displacement measurement.

Supplementary Table S5: Due to the large size, this table is part o the Data repository. Measurements of displacement for the 1999 M_w 7.1 Hector Mine earthquake. Column 1 and 2 contains gives the location of the displacement measurements from the stacked profiles in Latitude and Longitude, column 3 details the displacement and column 4 the 1σ uncertainty of the displacement measurement.

SOM References

40. Milliner, C.W., et al. Quantifying near-field and off-fault deformation patterns of the 1992 Mw 7.3 Landers earthquake. *Geochem.Geophys.Geosyst.*(2015).

41. Okubo, P.G., Aki, K. Fractal geometry in the San Andreas fault system. *Journal of Geophysical Research: Solid Earth (1978–2012)* **92,** 345-355 (1987).

42. Cello, G. Fractal analysis of a Quaternary fault array in the central Apennines, Italy. *J.Struct.Geol.* **19,** 945-953 (1997).
